# Retrospective Comparison of Commercially Available Automated Insulin Delivery With Open-Source Automated Insulin Delivery Systems in Type 1 Diabetes

**DOI:** 10.1177/19322968241230106

**Published:** 2024-02-16

**Authors:** Anna Schütz, Birgit Rami-Merhar, Ingrid Schütz-Fuhrmann, Nicole Blauensteiner, Petra Baumann, Tina Pöttler, Julia K. Mader

**Affiliations:** 1 Department of Pediatric and Adolescent Medicine, Medical University of Vienna, Vienna, Austria; 2 Karl Landsteiner Institute, Endocrinology and Nephrology, Vienna, Austria; 3 Department of Endocrinology and Nephrology, Clinic Hietzing, Vienna Health Care Group, Vienna, Austria; 4 Division of Endocrinology and Diabetology, Department of Internal Medicine, Medical University of Graz, Graz, Austria

**Keywords:** automated insulin delivery, hybrid closed loop, open source, type 1 diabetes mellitus

## Abstract

**Background:**

Automated insulin delivery (AID) systems have shown to improve glycemic control in a range of populations and settings. At the start of this study, only one commercial AID system had entered the Austrian market (MiniMed 670G, Medtronic). However, there is an ever-growing community of people living with type 1 diabetes (PWT1D) using open-source (OS) AID systems.

**Materials and Methods:**

A total of 144 PWT1D who used either the MiniMed 670G (670G) or OS-AID systems routinely for a period of at least three to a maximum of six months, between February 18, 2020 and January 15, 2023, were retrospectively analyzed (116 670G aged from 2.6 to 71.8 years and 28 OS-AID aged from 3.4 to 53.5 years). The goal is to evaluate and compare the quality of glycemic control of commercially available AID and OS-AID systems and to present all data by an in-depth descriptive analysis of the population. No statistical tests were performed.

**Results:**

The PWT1D using OS-AID systems spent more time in range (TIR)_70-180 mg/dL_ (81.7% vs 73.9%), less time above range (TAR)_181-250 mg/dL_ (11.1% vs 19.6%), less TAR_>250 mg/dL_ (2.5% vs 4.3%), and more time below range (TBR)_54-69 mg/dL_ (2.2% vs 1.7%) than PWT1D using the 670G system. The TBR_<54 mg/dL_ was comparable in both groups (0.3% vs 0.4%). In the OS-AID group, median glucose level and glycated hemoglobin (HbA1c) were lower than in the 670G system group (130 vs 150 mg/dL; 6.2% vs 7.0%).

**Conclusion:**

In conclusion, both groups were able to achieve satisfactory glycemic outcomes independent of age, gender, and diabetes duration. However, the PWT1D using OS-AID systems attained an even better glycemic control with no clinical safety concerns.

## Background

The majority of people living with type 1 diabetes (PWT1D) are still unable to achieve optimal glycemic control when performing insulin self-titration.^1-4^ Intensive research is focused on glucose-responsive insulin delivery aiming to help PWT1D to meet their treatment goals and to enhance their overall quality of life and life expectancy.^5-7^ Over the recent decades, ample research has been spent on the development of automated insulin delivery (AID) systems.^6-8^ An AID system combines a continuous glucose monitoring (CGM) system, an insulin infusion pump, and a glucose control algorithm to adjust basal insulin automatically according to the insulin requirements based on the real-time CGM data.^6,8,9^

With the approval of the first commercial AID system (MiniMed 670G system, Medtronic, Northridge, CA, USA) in 2016, the first AID system was introduced to the market and the clinical practice in 2017.^6,10^ Studies showed that the 670G system was safe to use^11^ and can improve glycemic control by increasing time in range (TIR) and reducing glycated hemoglobin (HbA1c) in adults and children with type 1 diabetes (T1D).^12-16^

Despite technological and commercial advancements in the field of diabetes care, PWT1D may believe that innovation and regulation are moving slowly. As a result, open-source AID (OS-AID) systems, with various control algorithms, features, and hardware compatibility, have been developed by PWT1D outside the usual regulatory environment.^17-20^ OpenAPS, AndroidAPS, and Loop are the three most widely used OS-AID systems.^21^

The PWT1D can construct their own AID system to close their loop between their commercially available insulin pump and CGM system.^17-19^ Establishing and programming OS-AID systems involves a significant amount of effort, experience, and time. Their use is generally confined to a unique group of highly chosen expert tech users.^22^ Evidence suggests that OS-AID systems can safely improve glycemic control and quality of life in adults and children with T1D.^20,21,23-26^

To our knowledge, only one similar, retrospective analysis has been performed, including 68 adults, to explore differences in user characteristics, safety, and efficacy between OS-AID and commercially available AID systems. Jeyaventhan et al^27^ observed a significantly improved change in HbA1c (*P* = .004) and in TIR_70-180 mg/dL_ (*P* = .024) in adults with T1D using OS-AID compared with 670G.

## Materials and Methods

The multicenter, retrospective study was conducted at the Department of Pediatric and Adolescent Medicine (Medical University of Vienna, Vienna, Austria), at the Department of Endocrinology and Nephrology (Clinic Hietzing, Vienna Health Care Group, Vienna, Austria), and at the Department of Endocrinology and Diabetology (Medical University of Graz, Graz, Austria). The research protocol was approved by the ethics committee of the Medical University of Vienna (ethics committee no. 1068/2020), the ethics committee of the City of Vienna (ethics committee no. 21-021-VK, 21-022-VK) and the ethics committee of the Medical University of Graz (ethics committee no. 33-254 ex 20/21).

The PWT1D who used either the commercially available AID system (MiniMed 670G system, Medtronic) or OS-AID systems routinely for a period of at least three to a maximum of six months between February 18, 2020 and January 15, 2023 were retrospectively analyzed. At the time of participant selection, the MiniMed 670G system was the only commercially available AID system in Austria. The abbreviation “670G” will be used for MiniMed 670G system users and the abbreviation “OS-AID” for OS-AID system users.

The key inclusion criterion was a diagnosis of T1D. The key exclusion criteria were pregnancy, diabetes mellitus other than T1D, and a diabetes duration less than six months.

Real-world clinical data of the 670G system users were obtained from the CareLink system and of the OS-AID system users from the Internet platform Nightscout (Nightscout Foundation, Littleton, CO, USA), the data hub and processor xDrip+ (Nightscout Foundation, Littleton, CO, USA), the diabetes therapy management software Tidepool (Tidepool, Palo Alto, CA, USA) and Dexcom CLARITY (Dexcom, Inc., San Diego, CA, USA). Further data (eg, age, gender) were collected from the (electronic) medical records and were documented in an Excel sheet in a pseudonymous manner. Due to the retrospective nature of the study, baseline data (eg, HbA1c) were not available at standardized time points. Therefore, data, such as HbA1c, height, and weight were collected within the selected study period of each individual. Data, such as age and diabetes duration, were calculated in the beginning of the study period. Eventually, 116 670G users aged from 2.6 to 71.8 years and 28 OS-AID users aged from 3.4 to 53.5 years were included in the analysis. Although the 670G system is approved for adults and children greater than or equal to seven years,^14^ children aged below seven years were included in this study. During the selection process, 112 PWT1D (97 on 670G and 15 on OS-AID) had to be excluded. The reasons for the exclusions were discontinuation, pregnancy, system use shorter than three months, diabetes duration less than six months, and unavailable CGM data.

The aim of this study is to assess the ability of the at the time only commercially available AID system (670G) to maintain sensor glucose (SG) levels within the target range of 70 to 180 mg/dL in comparison with OS-AID systems in PWT1D, including children, adolescents, and adults. The focus is to assess and compare the quality of glycemic control of 670G and OS-AID systems and to evaluate the safety of 670G and OS-AID systems in terms of episodes and severity of hypoglycemia and frequency of diabetic ketoacidosis (DKA).

The primary endpoint is the difference between the two groups in time spent in the target range (TIR_70-180 mg/dL_), in percentage, during the analysis period of at least three to a maximum of six months.

The secondary outcomes include time spent above target glucose (time above range [TAR]_>181-250 mg/dL_), time spent above target glucose (TAR_>250 mg/dL_), time spent below glucose target (time below range [TBR]_54-69 mg/dL_), and time spent below target glucose (TBR_<54 mg/dL_). Based on the Advanced Technologies & Treatments for Diabetes (ATTD) recommendations,^28^ the target range of 70 to 180 mg/dL was defined, along with TIR_70-180 mg/dL_ of >70%, TBR_<70 mg/dL_ of <4%, TBR_<54 mg/dL_ of <1%, TAR_>180 mg/dL_ of <25%, and TAR_>250 mg/dL_ of <5%.

In addition, the interrelation between TIR/TBR/TAR and gender, diabetes duration (<1, 1-≤5, >5-≤10, and >10 years), and different age groups were evaluated, respectively. The age groups (<13, ≥13-<26, ≥26, and ≥60 years) are selected based on a study of Foster et al^29^ and Boughton et al.^30^

Additional secondary outcomes include HbA1c, glucose management indicator (GMI), median glucose levels, coefficient of variation (CV), and glycemia risk index (GRI). The GRI has recently been described as a novel composite metric calculated from TBR_<70 mg/dL_, TBR_<54 mg/dL_, TAR_>180 mg/dL_, and TAR_>250 mg/dL_ to assess of the quality of glycemic control according to the risk for hypoglycemia and hyperglycemia.^31^ Based on the American Diabetes Association (ADA)^32^ and International Society for Pediatric and Adolescent Diabetes (ISPAD)^33^ recommendations, an HbA1c target of <7.0% (53.0 mmol/mol) was defined. The CV target of ≤36% was defined based on the study of Monnier et al.^34^ The GRI was calculated based on the study of Klonoff et al.^31^

Moreover, the frequency of severe hypoglycemic events requiring third-party help, frequency of DKA, diabetes-related complications (retinopathy, nephropathy, peripheral neuropathy, diabetic foot syndrome [DFS], acute myocardial infarction [AMI], peripheral artery disease [PAD], and stroke), and comorbidities (hypertension, hyperlipidemia) were analyzed.

Statistical analyses were performed using IBM SPSS Version 27 (SPSS, Armonk, NY, USA). Due to the different group sizes, n=116 (670G) and n=28 (OS-AID), the focus is to present all PWT1D data by an in-depth description of the population and no statistical tests are performed.

## Results

### Baseline Characteristics

A total of 144 PWT1D were included in the analysis, n=116 (670G) and n=28 (OS-AID). The baseline characteristics are summarized in Table 1.

**Table 1.** Baseline Characteristics Shown by Treatment Group. Data Given in Median (Q1; Q3).

	Valid n	670G	Valid n	OS-AID
Age (years)	116	32.3 [15.8; 50.6]	28	36.8 [30.2; 41.0]
Age range (years)		2.6-71.8		3.4-53.5
Age groups				
<13 years, No. (%)		22 (19.0)		2 (7.1)
13-26 years, No. (%)		28 (24.1)		2 (7.1)
≥26, No. (%)		66 (56.9)		24 (85.7)
Gender	116		28	
Female, No. (%)		52 (44.8)		14 (50.0)
Male, No. (%)		64 (55.2)		14 (50.0)
Body measurements for age group ≥18 years				
Height (cm)	83	174 [169; 180]	25	173 [169; 179]
Weight (kg)	83	78 [70; 91]	24	77 [66; 89]
BMI (kg/m^2^)	82	26 [24; 29]	24	24 [22; 28]
BMI-SDS for age group <18 years	32	0.4 [−0.2; 1.4]	3	0.2 [0.0; 0.8]
Diabetes duration (years)	116	16.6 [8.71; 28.1]	28	21.4 [12.7; 30.4]
Diabetes duration range (years)		0.5-67.9		1.8-42.2
System use for	116		28	
0-1 year, No. (%)		63 (54.3)		7 (25.0)
1-2 years, No. (%)		52 (44.8)		14 (50.0)
2-3 years, No. (%)		1 (0.9)		3 (10.7)
3-4 years, No. (%)		0 (0)		3 (10.7)
4-5 years, No. (%)		0 (0)		1 (3.6)
Insulin type				
Glulisine, No. (%)		6 (5.2)		2 (7.1)
Faster insulin aspart, No. (%)		1 (0.9)		3 (10.7)
Lispro, No. (%)		38 (33.0)		5 (17.9)
Aspart, No. (%)		70 (60.9)		18 (64.3)
HbA1c (%)	104	7.0 [6.7; 7.4]	21	6.2 [5.8; 6.4]
HbA1c (mmol/mol)		53.0 [49.7; 57.4]		44.3 [39.9;46.5]
GMI (%)	116	6.9 [6.7; 7.1]	28	6.4 [6.2; 6.7]
Glucose level (mg/dL)	116	150 [143; 158]	28	130 [122; 142]
Coefficient of variation (%)	116	33 [31; 36]	28	33 [30; 36]
GRI	116	29.7 [22.6; 39.0]	28	24.1 [15.0; 27.9]

Abbreviations: BMI-SDS, body mass index–standard deviation scores; GMI, glucose management indicator; GRI, glycemia risk index; HbA1c, glycated hemoglobin; OS-AID, open-source–automated insulin delivery.

The body mass index (BMI) was calculated for every user aged ≥18 years when both height and weight details were available. For each user aged <18 years, the age and gender-specific standard deviation score BMI-SDS was calculated.

The OS-AID users had a lower median HbA1c than 670G users. A total of 52 out of 104 670G users (50.0%) and 19 out of 21 OS-AID users (90.5%) met the HbA1c therapy goal of <7%. The median glucose level in the 670G group was higher than in the OS-AID group (150 mg/dL vs 130 mg/dL, −20 mg/dL). The median CV was comparable in both groups (33% vs 33%) and the majority of PWT1D in both groups achieved the CV target of ≤36% (670G: 67.0%, OS-AID: 75.0%).

During the study period, one event of DKA in one adult using 670G (while still in manual mode) and two severe hypoglycemic events requiring third-party help in two children using 670G were observed. A severe hypoglycemia occurred in a preschooler during automode; he was very tired and dizzy, but did not lose his consciousness nor did he have a seizure, but he needed help from his mother, therefore it was labeled as a case of severe hypoglycemia. Glucagon was not administered. The second severe hypoglycemia occurred in a teenager during an episode, where his sensor was not functioning, therefore he was not in automode. He also did not lose his consciousness, nor did he have a seizure, but he needed assistance. None of these events occurred in the OS-AID group. A stroke and two myocardial infarctions occurred in the 670G group. In all the three cases, they had a long diabetes duration of >20, >40, and >50 years, respectively, as well as additional risk factors such as hyperlipidemia and hypertension.

### Time in Different Target Ranges

**Figure 1.**
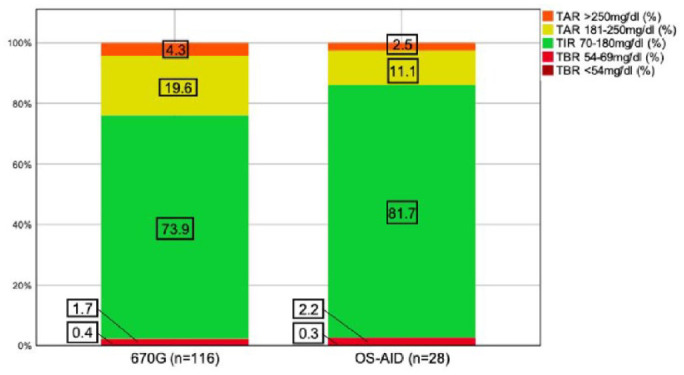
Histogram displaying median time in different ranges indicated by treatment group. Abbreviations: OS-AID, open-source–automated insulin delivery; TAR, time above range; TBR, time below range; TIR, time in range.

Compared with 670G users, the OS-AID users achieved a higher percentage of median TIR_70-180 mg/dL_ (81.7% vs 73.9%, −7.8%). A total of 62.1% (n=72) of 670G users and 96.4% (n=27) of OS-AID user met the TIR_70-180 mg/dL_ target of >70%. Both systems showed minimal time in hypoglycemia and the majority in both groups achieved the TBR_<70 mg/dL_ target of <4% (670G: 77.6%, OS-AID: 71.4%) and the TBR_<54 mg/dL_ target of <1% (670G: 75.0%, OS-AID: 85.7%). Approximately, half of 670G users and the majority of OS-AID users (92.9%) met the TAR_>180 mg/dL_ target of <25% (670G: 51.7%, OS-AID: 92.9%) and the TAR_>250mg/dl_ target of <5% (670G: 55.2%, OS-AID: 89.3%). A visualization of time in different ranges is given in Figure 1.

**Figure 2.**
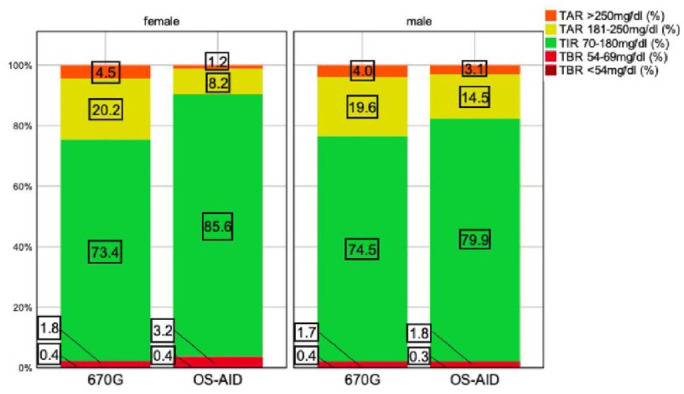
Histogram displaying median time in different ranges indicated by treatment group and gender. Abbreviations: OS-AID, open-source–automated insulin delivery; TAR, time above range; TBR, time below range; TIR, time in range.

Figure 2 visualizes the relationship between time in different ranges and gender. The best outcome in this cohort regarding TIR_70-180 mg/dL_ (85.6%), TAR_181-250 mg/dL_ (8.2%), and TAR_>250 mg/dL_ (1.2%) was noticed in female OS-AID users. However, they spent more time in TBR_<70 mg/dL_ (3.7%) and TBR_54-69 mg/dL_ (3.2%) than male OS-AID and 670G users.

**Figure 3.**
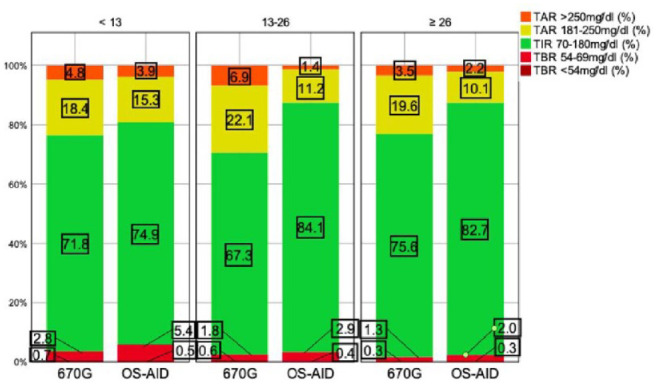
Histogram displaying median time in different ranges indicated by treatment group and age. Abbreviations: OS-AID, open-source–automated insulin delivery; TAR, time above range; TBR, time below range; TIR, time in range.

Figure 3 visualizes the relationship between time in different ranges and different age groups. The best outcome regarding TIR_70-180 mg/dL_, TAR_181-250 mg/dL_, and TAR_>250 mg/dL_ was observed in OS-AID users aged 13 to 26 years and ≥26 years. The greatest difference between 670G and OS-AID users regarding TIR_70-180 mg/dL_ (67.3% vs 84.1%), TAR_181-250 mg/dL_ (22.1% vs 11.2%), and TAR_>250 mg/dL_ (6.9% vs 1.4%) was noticed in the age group 13 to 26 years. The OS-AID users aged <13 years in this cohort spent most time in hypoglycemia compared with the other age groups (TBR_<70 mg/dL_ 5.9%, TBR_54-69 mg/dL_ 5.4%).

A subgroup of those ≥60 years of age was formed, which included only 10 670G users. The median TIR_70-180 mg/dL_ in the age group of ≥60 years was 76.3% and nine out of 10 users aged ≥60 years met the TIR _70-180 mg/dL_ target of >70%. All 670G users aged ≥60 years met the TBR_<70 mg/dL_ target of <4% and the TBR_<54 mg/dL_ target of <1%. Seven out of 10 users aged ≥60 years met the TAR_>180 mg/dL_ target of <25% and six out of 10 users aged ≥60 years did meet the TAR_>250 mg/dL_ target of <5%. According to GRI, both groups achieved good glycemic control (GRI values <40) with a tendency to better control for OS-AID versus MiniMed 670G (Table 1).

**Figure 4.**
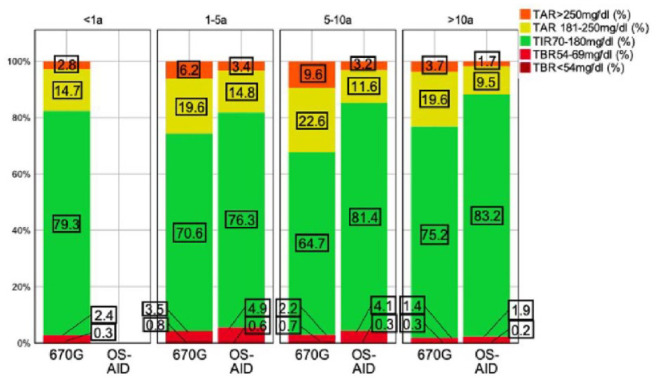
Histogram displaying median time in different ranges indicated by treatment group and diabetes duration. Abbreviations: OS-AID, open-source–automated insulin delivery; TAR, time above range; TBR, time below range; TIR, time in range.

Figure 4 visualizes the relationship between time in different ranges and diabetes duration. The best outcome regarding TIR_70-180 mg/dL_ (83.2%), TAR_181-250 mg/dL_ (9.5%), TAR_>250 mg/dL_ (1.7%), and TBR_<54 mg/dL_ (0.2%) was noticed in OS-AID users with a diabetes duration >10 years. The weakest outcome regarding TIR_70-180 mg/dL_ (64.7%) was observed in 670G users with a diabetes duration of 5 to 10 years.

In the supplemental material, we present data on summary of technology (type and generation) and algorithms used by the OS-AID group (Supplemental Table S1); diabetes-related complications, comorbidities, adverse events, and risk factors sor ted by treatment group (Supplemental Table S2); and summary of time in different target ranges sorted by treatment group (Supplemental Table S3).

## Discussion

Overall, these findings indicate a better glycemic outcome for OS-AID systems in comparison with 670G. The reported glycemic outcomes are consistent with prior observational studies conducted on OS-AID systems^35-37^ as well as the real-world for 670G.^38^ These findings align with the only other study comparing the OS-AID and commercially available AID systems.^27^ In comparison with Jeyaventhan et al’s study, both 670G and OS-AID users of this study achieved more TIR_70-180 mg/dL_ with less TBR_<70 mg/dL_, TBR_<54 mg/dL_, TAR_>180 mg/dL_, and TAR_>250 mg/dL_. The difference of improved TIR_70-180 mg/dL_ between 670G and OS-AID was lower than in the study of Jeyaventhan et al^24^ (−7.8% vs −10.3%).

As Jeyaventhan et al^27^ have already postulated, the reasons for the observed differences between systems are probably multifactorial. The PWT1D using OS-AID are a highly selective group who are possibly characterized by a greater degree of technological proficiency and more independent responsibility to assume for their own health, as well as better educational status compared with the general population with T1D.^20^

Although the use of Auto Mode is associated with improved glycemic control,^39^ a prospective observational study found that, after a year of use, 33% of the participants using the 670G system stopped using Auto Mode.^40^ The manufacturer recommends in their user guide to calibrate two to four times each day for using Auto Mode. In addition, in Auto Mode, the basal insulin delivery is automatically adjusted to maintain glucose levels to a standard SG target of 120 mg/dL.^41^ Sensor issues, including alarms for calibration, problems obtaining supplies, hypoglycemia fear, multiple daily injections preference, and sports were among the reasons for discontinuation. The study showed the significance of realistic expectations, usability, and human factors in ensuring Auto Mode use.^40^ The OS-AID users can define individual target ranges and no calibrations are needed, and thus, are more flexible compared with commercially available systems,^27^ which might be one of the various reasons for better glycemic control in OS-AID users. Furthermore, instead of using the recommended capillary glucose measurement, 670G users often use the SG value for calibration, which could lead to poorer glycemic control. However, the next generations of commercially available AID systems will no longer require mandatory calibrations.

However, studies suggest good glycemic control when using OS-AID systems. Advising the use of unapproved systems presents a number of challenging legal and ethical concerns that are difficult to resolve.^42^ Many health care providers (HCPs) are unsure how to effectively serve the PWT1D who use OS-AID systems in clinical care as OS-AID systems are unregulated.^20^ In contrast to commercial AID system manufacturers, there are no official onboarding programs (eg, instructional programs, customer hotline) for OS-AID users.^20^ Support for OS-AID system users to navigate the challenges of an unregulated system is mainly offered on a peer-to-peer basis and by informal mentoring roles, particularly through online channels such as Facebook groups.^43,44^ Ethical and legal concerns must be considered while employing an unregulated medical device, particularly the vulnerable group of children and adolescents because they are reliant on the knowledge and abilities of their caretakers.^36,45^ However, the MiniMed 670G system is approved for adults and children ≥7 years.^14^ Eight children aged <7 years using either the MiniMed 670G (n=7) or OS-AID (n=1) were included in this analysis. In both groups, these children used a system below the recommended age group or an unregulated system. Some might even argue that denying children aged <7 years access to these systems could be considered unethical as early use of technology is associated with improved glycemic control^46-48^ and evidence suggests that glycemic control improves with either commercially available AID or OS-AID in children aged ≤7 years.^15,16,26,49^

Wilmot and Danne^22^ point out that the risks of OS-AID compared with alternative treatment options are unknown as there are no randomized controlled trials on that subject. However, the consensus group believes that real-world data should also be considered in regulatory decisions as randomized controlled trials also have limitations in population diversity and reproduction in real-world clinical use.^20^ Nevertheless, evidence of OS-AID must be examined critically as most data are self-reported.^17,36,50,51^

Moreover, there is currently no treatment and no AID technology, which are either commercially available or open-sourced, capable of totally avoiding the acute complications that are associated with T1D, such as hypoglycemia, hyperglycemia, diabetic ketoacidosis, seizure, coma, and death.^22^ In fact, although HCPs prioritize providing PWT1D with the best health information possible, most PWT1D only spend a few hours per year in consultation with their HCP. In addition, not all HCPs have the same level of expertise working with the most recent technologies. Therefore, self-management is essential and it is important to provide PWT1D with the most effective tools (education, technology, and equipment).^42^ Braune et al do not believe that OS-AID systems should be universally preferred over commercial alternatives, as they outline in their international consensus statement and practical guidance review. However, they encourage HCPs to address these systems as an alternative treatment option for PWT1D who may benefit from OS-AID systems.^20^ The role of HCPs is to respect autonomy of the PWT1D in their care while also ensuring that PWT1D and their caretakers can make educated decisions and understand the risks and advantages of their chosen option.^20,52^

In conclusion, both groups (either 670G or OS-AID) were able to achieve satisfactory glycemic outcomes independent of age, gender, and diabetes duration. However, PWT1D using OS-AID systems attained an even better glycemic control with no clinical safety concerns, despite spending slightly more time in hypoglycemia.

## Limitations and Bias

The contribution of this study rests on analysis of real-world data, covering a wide age range. The main limitation of this study is the vastly different group sizes, n=116 (670G) and n=28, (OS-AID), including the different group sizes in the various age and diabetes duration groups, leading to difficulties to compare these groups. However, statistical tests were not performed in this study and the two groups were only compared using descriptive statistics.

As already mentioned previously, the PWT1D using OS-AID are a very selected group of people. Thus, results seen in this population might not be transferable to people who use off-the-shelf devices, resulting in a potential selection bias. In addition, the heterogeneity of devices (sensors, pumps, and algorithms) used in OS-AID users might make interpretation of data difficult. For example, sensor accuracy may differ from different providers. However, as these products are commercially available, we believe it is justified to evaluate the glycemic parameters clinically.

The 670G users need to actively upload their real-world clinical data in the CareLink system on a regular basis. When 670G users do not use the CareLink system, the data are often lost; the unavailability of data for 58 PWT1D resulted in their not being included in this analysis. The 670G users who do not use this diabetes therapy management tool might not be as engaged with their diabetes and their diabetes therapy as needed, resulting in a potentially poor glycemic control.

In the OS-AID group, 14 PWT1D could not be included because of unavailable data. As OS-AID systems are highly individualized, data storage is different for each individual. Occasionally, it was unclear where the data were saved, and there was also no means to access them.

Another limitation is the lack of data for insulin and Auto Mode use as they could not be retrieved for either 670G or OS-AID in the available files. During the analysis of the available comma-separated values (CSV) files of 670G users from the CareLink system, we were experiencing several unforeseen difficulties. In the downloaded CSV file, the structure of columns changes within the document when a new row of variable names is included after some random amount of data. Data are not machine readable, due to their unpredictable shifts in structure, including random row changes and variable new column names. In addition, it is unclear how to retrieve insulin data from these CSV files. Although these CSV files are provided by the manufacturer, it was difficult to analyze data independent of the industry.

Due to the retrospective nature of the study, various data, especially the baseline characteristics, are incomplete. As the analysis period was during the COVID-19 pandemic and the majority of routine clinic visits were conducted over the phone or online, data (eg, HbA1c) were not available at standardized time points as during a randomized controlled trial.

## Supplemental Material

sj-docx-1-dst-10.1177_19322968241230106 – Supplemental material for Retrospective Comparison of Commercially Available Automated Insulin Delivery With Open-Source Automated Insulin Delivery Systems in Type 1 Diabetes Supplemental material, sj-docx-1-dst-10.1177_19322968241230106 for Retrospective Comparison of Commercially Available Automated Insulin Delivery With Open-Source Automated Insulin Delivery Systems in Type 1 Diabetes by Anna Schütz, Birgit Rami-Merhar, Ingrid Schütz-Fuhrmann, Nicole Blauensteiner, Petra Baumann, Tina Pöttler and Julia K. Mader in Journal of Diabetes Science and Technology
