# Supplementary material for: Retrospective Comparison of Commercially Available Automated Insulin Delivery With Open-Source Automated Insulin Delivery Systems in Type 1 Diabetes
Source: J Diabetes Sci Technol. 2024 Feb 16;19(4):1060–7. doi: 10.1177/19322968241230106 (PMC11571566; doi:10.1177/19322968241230106)
Supplement: sj-docx-1-dst-10.1177_19322968241230106 – Supplemental material for Retrospective Comparison of Commercially Available Automated Insulin Delivery With Open-Source Automated Insulin Delivery Systems in Type 1 Diabetes [file sj-docx-1-dst-10.1177_19322968241230106.docx]

**Supplemental Data**

Supplementary Table 1: Summary of technology (type and generation) and algorithms used by the OS-AID group.

|  | | Valid n | n | % |
| --- | --- | --- | --- | --- |
| Sensor |  | 28 |  |  |
|  | Dexcom G5 |  | 3 | 10.7% |
|  | Dexcom G6 |  | 23 | 82.1% |
|  | Free Style Libre |  | 1 | 3.6% |
|  | Libre 2 |  | 1 | 3.6% |
| Pump |  | 28 |  |  |
|  | Accu-Chek Combo |  | 6 | 21.4% |
|  | Accu-Chek Insight |  | 3 | 10.7% |
|  | Dana RS |  | 2 | 7.1% |
|  | Minimed Paradigm 522 |  | 1 | 3.6% |
|  | Minimed Paradigm 722 |  | 2 | 7.1% |
|  | Minimed Veo 754 |  | 1 | 3.6% |
|  | Omnipod |  | 13 | 46.4% |
| Algorithm |  | 28 |  |  |
|  | AAPS |  | 11 | 39.3% |
|  | Loop |  | 14 | 50.0% |
|  | OpenAPS |  | 3 | 10.7% |

Supplementary Table 2: Diabetes-related complications, comorbidities, adverse events and risk factors sorted by treatment group.

|  | | 670G | | OS-AID | |
| --- | --- | --- | --- | --- | --- |
|  |  | Valid n | % | Valid n | % |
| DKA | No | 115 | 99.1 | 25 | 100 |
|  | Yes | 1 | 0.9 | 0 | 0 |
| Severe hypoglycemia | No | 114 | 98.3 | 25 | 100 |
|  | Yes | 2 | 1.7 | 0 | 0 |
| Retinopathy | No | 94 | 87.0 | 22 | 91.7 |
|  | Yes | 14 | 13.0 | 2 | 8.3 |
| Nephropathy | No | 102 | 92.7 | 25 | 100 |
|  | Yes | 8 | 7.3 | 0 | 0 |
| Peripheral neuropathy | No | 110 | 94.8 | 25 | 100 |
|  | Yes | 6 | 5.2 | 0 | 0 |
| PAD | No | 116 | 100 | 25 | 100 |
|  | Yes | 0 | 0 | 0 | 0 |
| Amputations | No | 116 | 100 | 25 | 100 |
|  | Yes | 0 | 0 | 0 | 0 |
| DFS | No | 116 | 100 | 25 | 100 |
|  | Yes | 0 | 0 | 0 | 0 |
| Stroke | No | 115 | 99.1 | 25 | 100 |
|  | Yes | 1 | 0.9 | 0 | 0 |
| AMI | No | 114 | 98.3 | 25 | 100 |
|  | Yes | 2 | 1.7 | 0 | 0 |
| Hypertension | No | 84 | 72.4 | 21 | 84.0 |
|  | Yes | 32 | 27.6 | 4 | 16.0 |
| Hyperlipidemia | No | 75 | 64.7 | 21 | 84.0 |
|  | Yes | 41 | 35.3 | 4 | 16.0 |
| Smoking | No | 107 | 94.7 | 24 | 96.0 |
|  | Yes | 6 | 5.3 | 1 | 4.0 |

Supplementary Table 3: Summary of time in different target ranges sorted by treatment group.

|  | 670G | | | OS-AID | | |
| --- | --- | --- | --- | --- | --- | --- |
|  | Median | Q1 | Q3 | Median | Q1 | Q3 |
| TIR_70-180mg‎/dl_ (%) | 73.9 | 66.0 | 78.7 | 81.7 | 76.2 | 88.5 |
| TBR_<70mg‎/dl_ (%) | 2.2 | 1.0 | 3.9 | 2.5 | 1.4 | 4.4 |
| TBR_54-69mg‎/dl_ (%) | 1.7 | 0.9 | 2.8 | 2.2 | 1.3 | 4.1 |
| TBR_<54mg‎/dl_ (%) | 0.4 | 0.1 | 1.0 | 0.3 | 0.2 | 0.6 |
| TAR_>180mg‎/dl_ (%) | 24.5 | 19.7 | 31.6 | 13.8 | 8.1 | 20.3 |
| TAR_181-250mg/dl_ (%) | 19.6 | 16.0 | 23.1 | 11.1 | 7.5 | 15.9 |
| TAR_>250mg‎/dl_ (%) | 4.3 | 2.4 | 7.4 | 2.5 | 0.5 | 3.4 |
